# Intermuscular Coherence in Spinocerebellar Ataxias 3 and 6: a Preliminary Study

**DOI:** 10.1007/s12311-023-01585-7

**Published:** 2023-07-10

**Authors:** Naoum P. Issa, Serdar Aydin, Shail Bhatnagar, Nicholas W. Baumgartner, Jacquelyn Hill, Sravya Aluri, Chloe S. Valentic, Eric Polley, Christopher M. Gomez, Kourosh Rezania

**Affiliations:** 1 Department of Neurology, University of Chicago, 5841 S. Maryland Ave., MC2030, Chicago, IL 60637, USA; 2 Purdue University, West Lafayette, IN, USA; 3 Michigan State University, East Lansing, MI, USA

**Keywords:** Spinocerebellar ataxia, Intermuscular coherence, Biomarker, SCA3, SCA6, Neurodegenerative disease, Beta-gamma frequency

## Abstract

Spinocerebellar ataxias (SCAs) are familial neurodegenerative diseases involving the cerebellum and spinocerebellar tracts. While there is variable involvement of corticospinal tracts (CST), dorsal root ganglia, and motor neurons in SCA3, SCA6 is characterized by a pure, late-onset ataxia. Abnormal intermuscular coherence in the beta-gamma frequency range (IMCβγ) implies a lack of integrity of CST or the afferent input from the acting muscles. We test the hypothesis that IMCβγ has the potential to be a biomarker of disease activity in SCA3 but not SCA6. Intermuscular coherence between biceps brachii and brachioradialis muscles was measured from surface EMG waveforms in SCA3 (*N* = 16) and SCA6 (*N* = 20) patients and in neurotypical subjects (*N* = 23). IMC peak frequencies were present in the β range in SCA patients and in the γ range in neurotypical subjects. The difference between IMC amplitudes in the γ and β ranges was significant when comparing neurotypical control subjects to SCA3 (*p* < 0.01) and SCA6 (*p* = 0.01) patients. IMCβγ amplitude was smaller in SCA3 patients compared to neurotypical subjects (*p* < 0.05), but not different between SCA3 and SCA6 patients or between SCA6 and neurotypical subjects. IMC metrics can differentiate SCA patients from normal controls.

## Introduction

Spinocerebellar ataxias (SCAs) are a group of familial neurodegenerative diseases that involve the cerebellum and the brainstem, with their most common clinical presentation being a progressive ataxia. The most common type of SCA in the USA and worldwide is SCA3, followed by SCA2 and SCA6 [1], with SCA3 and SCA6 together accounting for more than a third of familial ataxias [2]. SCA3 has an onset in childhood to mid-adulthood with heterogeneous clinical manifestations, including pyramidal and extrapyramidal features, amyotrophy, and peripheral neuropathy, in addition to ataxia [3]. SCA6, on the other hand, is primarily an ataxic disorder with a mean age of onset of 52 years [1, 4]. Classically, diagnosis of the SCA subtype has relied on clinical presentation, with definitive diagnosis now done by genetic testing.

There has been substantial expansion in therapeutic approaches for inherited neurodegenerative diseases with targeted treatments including antisense oligonucleotides and stem cell-based approaches in the preclinical stage for different types of SCA [5]. As these therapies come to clinical trials, there will be a need for biomarkers of degeneration in central and peripheral pathways involving the cerebellum, pyramidal system, and sensory pathways in symptomatic and presymptomatic stages of SCA. Although clinical outcome measures are commonly used to capture the severity of symptoms in SCAs, an increasing number of imaging and fluid biomarkers are being identified for future clinical trials [6]. The Scale for the Assessment and Rating of Ataxia (SARA) is a validated clinical scale used for monitoring the progression of ataxia [4, 7]. SARA has rather low effect sizes in longitudinal studies, therefore necessitating a large sample size to detect changes in progression, which is difficult in SCAs given their rarity [8]. For example, in a large European cohort, the rate of increase of SARA was steepest in SCA1 and only ~4%/year for SCA3 [9]. SARA is, therefore, not an ideal tool for monitoring disease progression in clinical trials, which typically last 6–12 months. Certain MRI sequences such as volumetric analysis of the cerebellar cortex, superior cerebellar peduncle and brainstem, diffusion tensor imaging, and tractography have shown promising results in longitudinal studies on SCA [6, 8], but these are not validated and available only in a limited number of centers. A combination of neurophysiological methods, including nerve conduction studies (NCS), somatosensory evoked potentials (SEPs), and motor cortical evoked potentials using transcranial magnetic stimulation (TMS), are reliable methods for assessment of peripheral nerves, dorsal root ganglia, and corticospinal tracts [10]. However, these tests usually cannot be done on a repetitive basis in the setting of a clinical trial because of the associated discomfort and—in the case of TMS—lack of widespread availability. Ideal biomarkers to objectively monitor SCA disease progression are, therefore, not yet available.

Coherence analysis has been extensively used to study the integrity of pathways involved in the performance of a motor act. Coherence in neurophysiological signals measured from the sensorimotor cortex, subcortical regions, and lower motor neurons represents the synchronization of neuronal activity in these spatially distant structures involved in a coordinated movement [11]. Corticomuscular coherence (CMC) in the β frequency range (13–30 Hz) is present between oscillatory activity of the motor cortex and active motor units in the contralateral limbs [12–16]. The cortically originated signals can also be assessed by measuring the coherence between co-activated muscles (intermuscular coherence; IMC) [12, 14, 17, 18]. IMC in the β and γ frequency ranges becomes abnormal in diseases involving upper motor neurons including primary lateral sclerosis and amyotrophic lateral sclerosis (ALS) [19, 20], and also by sensory deafferentation of the acting muscles. Such changes could be the result of dysfunction at the level of the peripheral nerves, dorsal root ganglia, or dorsal columns [21, 22]. Determination of IMC or CMC is, therefore, a potential biomarker not only for motor neuron diseases but also for other diseases of the nervous system that affect the motor system such as SCAs.

In this study, we apply a method for assessing IMC using a conventional electromyography (EMG) apparatus [20] to determine if IMC has the potential as a new biomarker for SCA3 or SCA6. In previous work by our group [20], we found that IMC between the biceps brachii and brachioradialis muscles had a consistent peak in the frequency range between 20 and 40 Hz in neurotypical control subjects and that IMC in this frequency range best-distinguished patients with upper motor neuron dysfunction due to ALS from neurotypical controls. Because the 20–40 Hz frequency range crosses the boundary between β and γ frequency bands, we use the term IMCβγ to refer to the average IMC between 20 and 40 Hz. In this study, we test the hypothesis that IMCβγ would be abnormal in SCA3 patients, who often have significant involvement in corticospinal tract and/or sensory pathways.

## Methods

This is a cross-sectional study on a cohort of patients with SCA3 (*N* = 16; age in years [mean ± standard deviation] 54.7 ± 14.6), SCA6 (*N* = 20; age 63.3 ± 9.5), and neurotypical control subjects (*N* = 23; age 46.8 ± 15.9). Subjects were excluded from the study if they were <18 years, unable to provide informed consent, or were unable to perform the study task. The data collected included demographic information, symptom duration, results of the genetic testing (CAG trinucleotide expansion size), the Scale for the Assessment and Rating of Ataxia (SARA), which ranges from 0 (no cerebellar symptoms) to 40 (most severe cerebellar symptoms) to measure the severity of ataxia [7], and assessment of IMC as previously described [20]. The study was approved by the Institutional Review Board of the University of Chicago Biological Science Division, and subjects provided written informed consent before research-related data were collected.

### Intermuscular Coherence Measurements

Surface EMG was recorded simultaneously from the biceps brachii and brachioradialis muscles from the dominant arm. Bipolar surface EMG electrodes with a 3 cm spacing (Nicolet bipolar disk bar electrodes; Natus Medical, Inc., Pleasanton, California) were placed over the muscle bellies, and a ground electrode was placed on the volar surface of the wrist. The nearest contacts on the two different muscles were separated by at least 8 cm.

Muscle activity was recorded while the subject held their forearm parallel to the ground and perpendicular to the upper arm. The arm was at their side, with the shoulder adducted and neutrally rotated. Recording sessions consisted of three epochs each lasting 30 s and separated from each other by 30 s of rest with the hand in a semi-pronated position [20]. Surface EMG signals were acquired using a Cadwell EMG system (model Sierra-Summit, Cadwell Industries, Inc., Kennewick, WA) with a high-pass filter of 5 Hz, a low-pass filter of 200 Hz, a notch filter at 60 Hz, and at a sampling rate of 64,000 samples/second.

Signals were down-sampled to 4267 Hz using the Matlab decimate function prior to the calculation of IMC. Surface EMG activity was segmented into blocks of ~0.25 s. The IMC calculation was implemented in Matlab using the mscohere (magnitude-squared coherence) algorithm. There was no overlap in segments. The confidence limit is the coherence level below which coherence is not statistically different from zero. The confidence limit is defined as follows: *CL* = 1 – *α*^1/(*S* – 1)^ in which *α* is the desired significance level (0.05) and *S* is the number of segments analyzed [23].

Coherence is defined for stationary signals, meaning changes in load or arm movement during the task would degrade intermuscular coherence calculations. This was especially an issue in patients with tremor, for whom the more frequent arm movements could artificially reduce coherence measures. We used the same process as in Issa et al. 2017 [20] to exclude segments of data that violate stationarity, with modifications to reduce the amount of data excluded, set a maximum allowable variance, and ensure a low confidence limit. The exclusion process was iterative, with the goal of having the coefficient of variation (COV) for root mean squared (RMS) amplitudes (standard deviation of block RMS amplitudes/mean of block RMS amplitudes) be smaller than 0.15. Individual 0.25-s blocks of EMG data that had an RMS amplitude greater than four standard deviations from the mean RMS amplitude of a subject’s EMG data set were initially removed. If this post hoc processing step did not achieve a COV below 0.15, then blocks with progressively smaller deviations from the mean were removed until the COV was less than 0.15. Only subjects in whom a sufficient number of segments were retained to provide an IMC confidence limit below 0.03 were included in the final analysis. The average number of segments retained was 221 ± 88 (mean ± standard deviation) for the neurotypical group, 224 ± 88 for the SCA3 group, and 211 ± 61 for the SCA6 group.

The average IMC was calculated in specific frequency bands. IMC averaged between 20 and 40 Hz (IMCβγ) has been identified as a potential marker for upper motor neuron dysfunction in the biceps brachii-brachioradialis muscle pair [20]. This frequency range crosses the boundary between the β-band and γ-band but does not encompass the full β- or γ-bands. Average IMC was also calculated in different frequency ranges, including between 30 and 40 Hz and between 15 and 25 Hz.

### Statistical Analysis

Stata version SE17 (StataCorp LLC, College Station, TX) was used for statistical analysis. Continuous data were presented with mean and standard deviation (SD) and categorical data as number and percentile. Comparisons of age, SARA score, disease duration, and IMC amplitudes were done using the Mann-Whitney U test, and gender and handedness were compared with a test of proportions. The associations between CAG trinucleotide expansion size and IMC metrics were assessed with linear regression. Alpha was set to 0.05. The Bonferroni-Holm method was used to adjust *p*-values for multiple comparisons of IMC measures [24], with correction for two different IMC measures (IMCβγ and IMC_30–40_ – IMC_15–25_). Receiver-operating characteristic (ROC) curves were calculated to assess the diagnostic ability of IMC metrics to differentiate subtypes of SCA from each other and from neurotypical subjects [25]. An area under the ROC curve (AUC) of 0.9–1.0 was considered excellent, 0.8–0.9 good, 0.7–0.8 fair, 0.6–0.7 poor, and between 0.5 and 0.6 non-diagnostic.

## Results

Intermuscular coherence was measured in patients with SCA3 (*N* = 16), SCA6 (*N* = 20), and in neurotypical control subjects (*N* = 23). Group characteristics are shown in Table 1. All pairwise comparison results are shown in supplemental tables 1–2. Gender and handedness proportions across the three study groups and disease duration among the SCA subgroups were not significantly different (*p* > 0.05 for all comparisons; Supplemental Tables 1 and 2). SCA6 subjects were significantly older than the neurotypical subjects (*p* = 0.002), but not the SCA3 group (*p* = 0.10), and the average age of the SCA3 group was not different from the neurotypical control group (*p* = 0.12). The difference in SARA scores between the SCA6 and SCA3 groups was not statistically significant (*p* = 0.07).

**Fig. 1:**
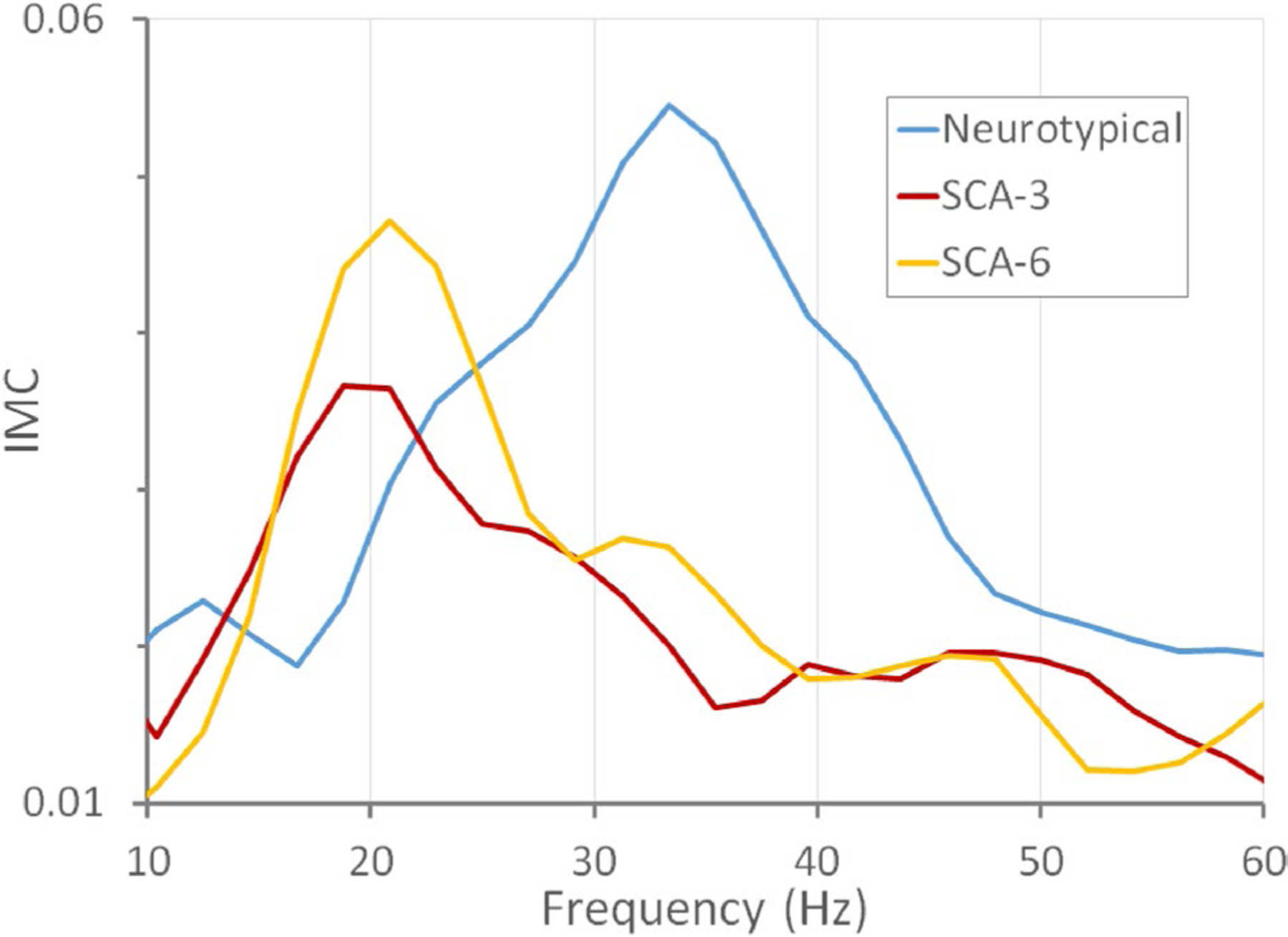
Average intermuscular coherence (IMC) profiles as a function of frequency for neurotypical control subjects and patients with SCA3 and SCA6

Intermuscular coherence varied with frequency in all measured groups (Fig. 1). The profile for neurotypical controls shows a peak in coherence between 20 and 40 Hz, similar to that previously reported for an independent set of neurotypical subjects [20].

**Fig. 2:**
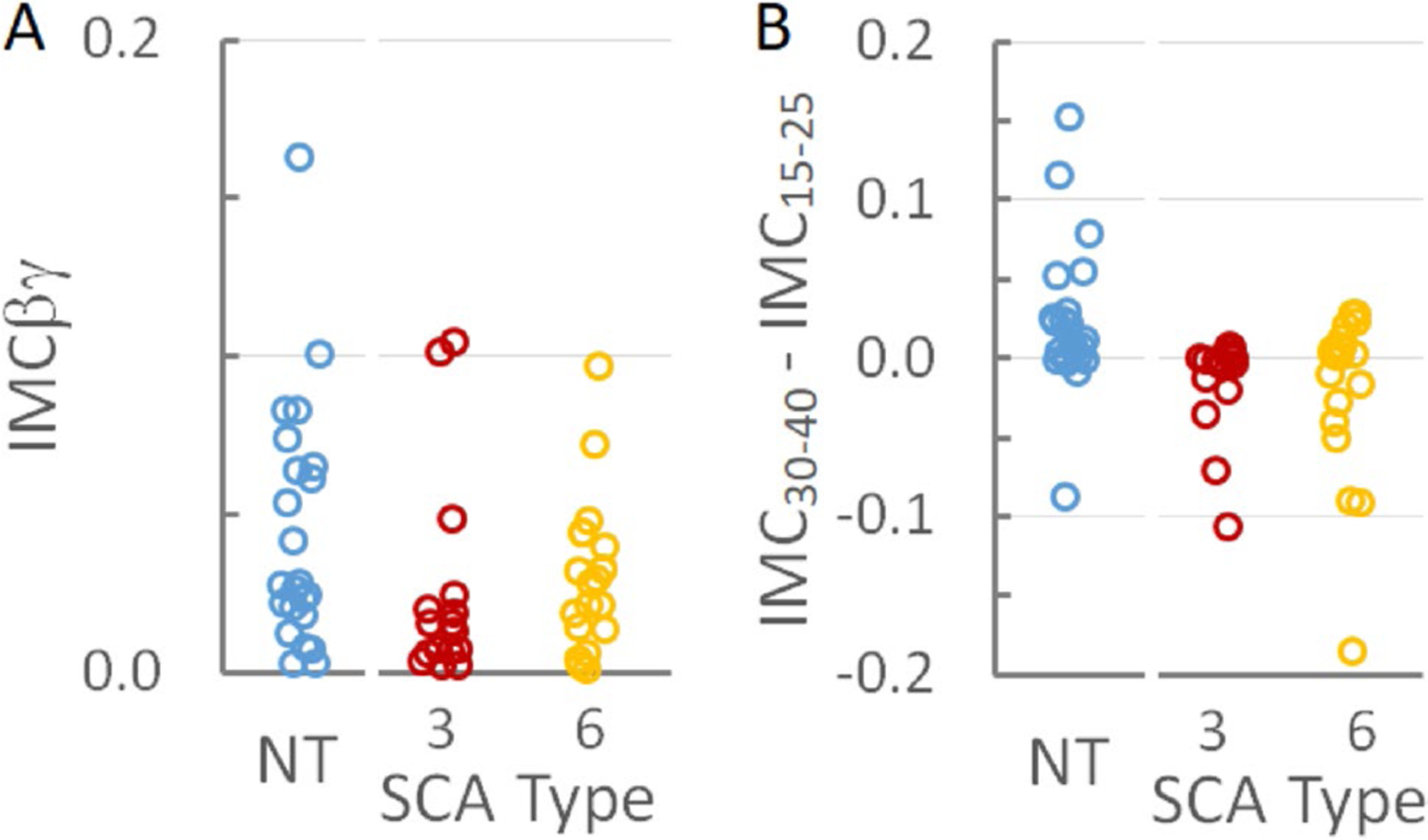
IMC in individual subjects. **A** Distribution of IMCβγ by SCA type and in neurotypical (NT) control subjects. IMCβγ was larger in neurotypical subjects than in SCA3 patients, but there was no difference in IMCβγ between SCA6 patients and either neurotypical subjects or SCA3 patients. **B** The distribution of IMC_30–40_ – IMC_15–25_ by SCA type and in neurotypical control subjects. IMC_30–40_ – IMC_15–25_ was larger in neurotypical subjects than in either SCA3 or SCA6 patients

To determine if IMCβγ could help differentiate among the SCA subtypes or between patients with SCAs and neurotypical subjects,IMCβγ was measured for each subject. As suggested by the plots in Fig. 1, the average IMCβγ was smallest in SCA3 patients and largest in neurotypical control subjects (Table 1). When the distributions of individual IMCβγ were compared among groups (Fig. 2A), there was no significant difference between the IMCβγ amplitudes of SCA6 patients and neurotypical control subjects (*p* = 0.35; Supplemental Table 1). SCA3 patients had a significantly smaller IMCβγ than neurotypical subjects (*p* = 0.02, which remained significant after the Bonferroni-Holm correction for 2 comparisons; Supplemental Table 1), but there was no statistical difference between SCA3 and SCA6 patients (*p* = 0.17; Supplemental Table 2).

The IMC profiles for SCA3 and SCA6 appeared qualitatively different from those of neurotypical control subjects (Fig. 1) and different from previous findings from amyotrophic lateral sclerosis (ALS) subjects who showed a loss of coherence in the βγ range [20]. SCA3 and SCA6 subjects appeared to have a peak at lower frequencies than in the average neurotypical IMC profile. The difference between IMC at 30–40 Hz and IMC at 15–25 Hz was calculated for each subject to determine whether differences in the coherence peaks in the IMC profile could differentiate SCA3 or SCA6 from neurotypical subjects. The distributions of this difference (IMC_30–40_ – IMC_15–25_) were significantly different between neurotypical subjects and SCA3 patients (*p* = 0.001; *p* = 0.002 after the Bonferroni-Holm correction) and between neurotypical subjects and SCA6 patients (*p* = 0.015; *p* = 0.03 after the Bonferroni-Holm correction; Supplemental Table 1), but not between SCA3 and SCA6 groups (*p* = 0.46; Supplemental Table 2; Fig. 2B).

**Fig. 3:**
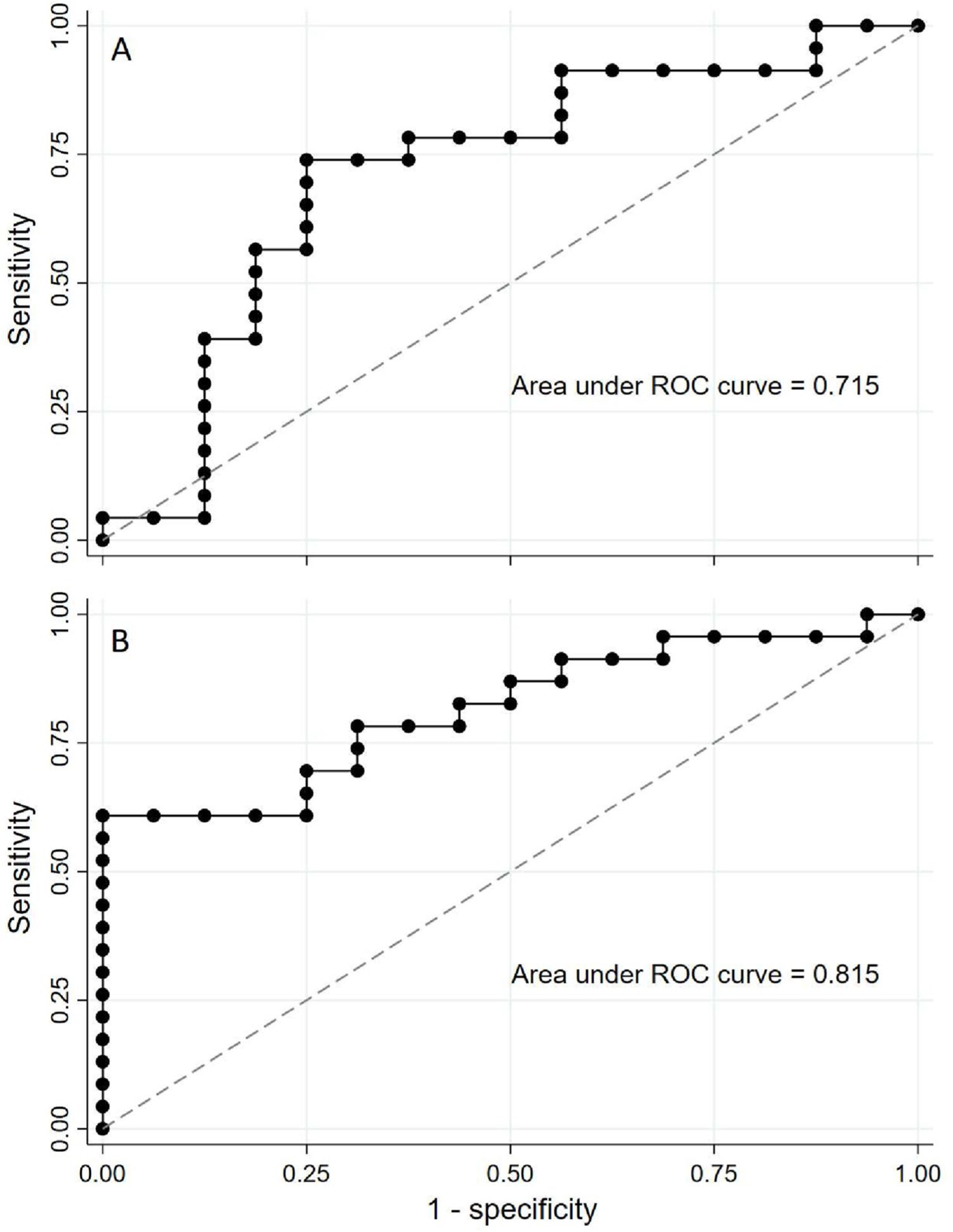
ROC curve SCA3 versus neurotypical control subjects. **A** IMCβγ provided fair ability (AUC 0.715) to differentiate SCA3 patients from neurotypical control subjects. **B** IMC_30–40_ – IMC_15–25_ provided good ability (AUC 0.815) to differentiate SCA3 patients from neurotypical control subjects

The ability of IMCβγ or IMC_30–40_ – IMC_15–25_ to differentiate patients with SCA3 or SCA6 from neurotypical subjects or the other SCA subtype was assessed using ROC analysis (Fig. 3). IMCβγ performed poorly in differentiating SCA6 from SCA3 or neurotypical controls (*AUC* < 0.7 for both comparisons; Supplemental Table 3). However, IMCβγ had fair diagnostic ability differentiating SCA3 patients from neurotypical subjects (*AUC* = 0.715; Supplemental Table 3, Fig. 3A). IMC_30–40_ – IMC_15–25_ had a different diagnostic performance than IMCβγ. While IMC_30–40_ – IMC_15–25_ could not differentiate between the two SCA subtypes (*AUC* = 0.57; Supplemental Table 4), it had fair diagnostic ability differentiating SCA6 from neurotypical controls (*AUC* = 0.72) and good ability in differentiating SCA3 from neurotypical controls (*AUC* = 0.82; Fig. 3B).

Linear regression was used to test whether IMC metrics were associated with the CAG triplet repeat number. For the SCA6 group, there was little variability in triplet repeat number (Table 1), with all but two patients having 22 repeats, one had 21 repeats, and one had 25 repeats. As would be expected based on the limited variability in the independent variable, neither IMCβγ (*p* = 0.85) nor IMC_30–40_ – IMC_15–25_ (*p* = 0.30) were correlated with triplet repeat number in the SCA6 group. For the SCA3 group, the triplet repeat number ranged between 62 and 77 (Table 1) but was not correlated with either IMCβγ (*p* = 0.88) or IMC_30–40_ – IMC_15–25_ (*p* = 0.95). There was, therefore, no significant association between triplet repeat number and IMC metrics in either SCA3 or SCA6 groups.

To determine whether IMCβγ or IMC_30–40_ – IMC_15–25_ have the potential to be markers for disease severity, they were compared to SARA scores and disease duration. SARA scores were strongly correlated with disease duration for SCA6 patients (*R*^2^ = 0.60, *p* = 0.00), but not for SCA3 patients (*R*^2^ = 0.02, *p* = 0.56). Neither SARA scores nor disease duration was correlated with IMCβγ or IMC_30–40_ – IMC_15–25_ in the SCA6 group (*p* > 0.3 for all comparisons). In SCA3 patients, neither IMC metric correlated with disease duration (*p* > 0.5 for all comparisons). While the correlation between SARA scores and IMCβγ was not statistically significant (*R*^2^ = 0.17, *p* = 0.11), SARA scores were significantly correlated with IMC_30–40_ – IMC_15–25_ for the SCA3 group (*R*^2^ = 0.27, *p* = 0.04). Therefore, IMC measures showed a signal of correlating with disease severity in SCA3 but not SCA6 patients.

## Discussion

Abnormally low IMCβγ is thought to represent a lack of integrity in the corticospinal tract or proprioceptive loss [14, 18, 19, 22, 26]. As these pathways are differently involved in the various SCA subtypes, we hypothesized that IMC could provide differentiating information between the subtypes or may serve as a biomarker for disease progression for certain SCAs.

The preliminary findings reported here suggest that IMCβγ is not significantly altered in patients with SCA6. This was the expected finding for SCA6 patients, in whom pathological changes are primarily cerebellar with minimal changes to the corticospinal system or peripheral nerves. Conversely, IMCβγ was significantly lower in SCA3 patients than in neurotypical control subjects, consistent with the mixed cerebellar, corticospinal, and peripheral nerve dysfunction found with SCA3.

While the main question addressed in this study was whether IMCβγ would be useful as a marker for SCA3 and/or SCA6, another characteristic of the IMC profile was identified as a good marker for SCAs. In both SCA3 and SCA6 groups, there was an IMC peak in the 15–25 Hz frequency range, with a comparatively lower peak in the 30–40 Hz frequency range. This pattern was qualitatively different from the IMC profile in neurotypical subjects and the pattern seen in ALS patients [20]. The difference between IMCs in the 30–40 Hz and 15–25 Hz bands (IMC_30–40_ – IMC_15–25_) differentiated the SCA subtypes from the neurotypical group better than IMCβγ did, with the best performance in differentiating SCA3 from neurotypical subjects having an area under the ROC curve of 0.82.

Our findings suggest that IMC metrics might be useful in monitoring disease progression in SCA3 but not SCA6. SARA scores were tightly coupled to SCA6 disease duration, consistent with the primacy of cerebellar symptoms in SCA6. Neither IMCβγ nor IMC_30–40_ – IMC_15–25_ were correlated with either SARA scores or SCA6 disease duration, suggesting that they are not good markers for purely cerebellar pathology. For SCA3 subjects, IMC_30–40_ – IMC_15–25_ was weakly correlated with disease severity as approximated by the SARA score. Interestingly, neither SARA scores nor IMC_30–40_ – IMC_15–25_ were correlated with disease duration for SCA3 patients. This is consistent with the findings of a previous study that did not find a significant correlation of disease duration as an independent variable with SARA score in SCA3 patients [4]. This might be because cerebellar dysfunction is only a part of the neurological impairment in SCA3. The correlation between SARA scores and IMC_30–40_ – IMC_15–25_ in SCA3 might be the result of a secondary correlation between SARA scores and corticospinal dysfunction. Future studies to address this issue should include a larger study group, longitudinal follow-up with repeated IMC measurements, and assessments of non-cerebellar symptoms in SCA, like the Inventory of Non-Ataxia Symptoms (INAS).

Previous studies have demonstrated the usefulness of coherence analysis in SCA. Velazquez-Perez, et al (2017) demonstrated abnormal β-band CMC in the lower limbs in 19 SCA2 patients, which correlated with prolongation of central motor conduction time, consistent with degeneration of the corticospinal tracts [27]. Abnormal CMC was present even in the absence of clinical evidence of corticospinal tract involvement in the aforementioned study. Other studies from the same investigators demonstrated lower IMCβ in symptomatic SCA2 patients as well as those in the prodromal state of the disease [28, 29].

Although the cerebellum plays a critical role in the timing of motor tasks [30], it is not clear if and how cerebellar output modifies CMC and IMC. Marsden et al (2000) found coherence in the 8–27 Hz range between the local field potentials of nucleus ventralis intermedius (VIM) of the thalamus (i.e., cerebellar thalamus), ipsilateral EEG, and contralateral surface EMG of the hand muscles in patients with isolated tremors [31]. The coherence between VIM and EMG activity was less apparent in patients with cerebellar disease. The EEG-EMG coherence was absent in the majority of the patients in that study, so it could not be determined if the cerebellar disease could affect the CMC.

The use of rectification in calculating intermuscular coherence has been heavily debated [28, 32–35], with most studies using rectified EMG. We chose not to rectify EMG signals because a previous study showed a clear difference between neurotypical subjects and ALS patients with upper motor neuron dysfunction using IMC from unrectified EMG signals [20] and because rectification has been shown to significantly attenuate IMC in the γ frequency band [28]. We also have confirmed that rectification abolishes the γ band coherence (data not shown), and thus, rectification could not be used to differentiate SCA patients from neurotypical subjects. Rectification has been suggested to result in distortion of the coherence in β to γ bands due to high levels of amplitude cancelations in those frequencies [35].

Given these preliminary findings, future studies will be able to address several limitations. The cross-sectional nature prevents the determination of whether IMC metrics change with time. In addition, future studies should include measures of non-cerebellar symptoms that might correlate with IMC better than does the SARA score. While significant correlations were found with a small number of the subjects, a larger sample size will be needed to both validate and better characterize the relationship between IMC and clinical findings in SCA patients. One potential confounder is that IMC was measured in only a single limb, while disease progression might be unequal across limbs. Subsequent studies could address this question by measuring IMC in all four limbs.

## Conclusion

In this proof of concept, single-center, and cross-sectional study, we demonstrated that IMC patterns are different in SCA3 and SCA6 patients compared to neurotypical control subjects. Both IMCβγ and IMC_30–40_ – IMC_15–25_ were able to distinguish SCA3 from control subjects, but IMC_30–40_ – IMC_15–25_ performed better than IMCβγ at identifying patients with SCA6. IMC, which can be done in a simple and inexpensive manner, is a potential biomarker for SCA3 and SCA6, but longitudinal studies will be needed to assess the viability of this biomarker.

## Supplementary Material

Supplementary tables 1-4 and Figure

## Figures and Tables

**Table 1:** Patient characteristics, including % female, age, handedness, SARA score, disease duration, and IMC values for neurotypical control subjects and SCA patients

	Neurotypical (*N* = 23)	SCA3 (*N* = 16)	SCA6 (*N* = 20)
Age mean in years (S.D.)	46.8 (15.9)	54.7 (14.6)	63.3 (9.5)
Age range (min-max)	22.5–70.0	30.0–74.7	44.8–85.5
% female	48%	63%	60%
% left handed	17%	19%	5%
SARA score (S.D.)	–	12.2 (5.9)	8.6 (6.8)
Disease duration in years (S.D.)	–	13.4 (12.6)	10.5 (9.3)
Triplet repeats (S.D.)	–	70.2 (4.4)	22.1 (0.7)
IMCβγ mean (S.D.)	0.044 (0.039)	0.024 (0.033)	0.030 (0.024)
IMC_30–40_ – IMC_15–25_ mean (S.D.)	0.022 (0.047)	−0.015 (0.032)	−0.020 (0.052)

*SARA* Scale for the Assessment and Rating of Ataxia, *S.D*. standard deviation, *IMCβγ* intermuscular coherence in the beta-to-gamma frequency range (20–40 Hz), *IMC*_*30–40*_ – *IMC*_*15–25*_ intermuscular coherence measured between 30 and 40 Hz minus intermuscular coherence measured between 15 and 25 Hz

## Data Availability

Anonymized data sets are available through request to the corresponding author.
