# Supplementary material for: Intermuscular Coherence in Spinocerebellar Ataxias 3 and 6: a Preliminary Study
Source: Cerebellum. Author manuscript; Available in PMC 2024 Apr 1. (PMC10776817; doi:10.1007/s12311-023-01585-7)
Supplement: Supplementary tables 1-4 and Figure [file NIHMS1944100-supplement-Supplementary_tables_1-4_and_Figure.docx]

Supplementary Materials

**SUPPLEMENTAL MATERIALS**

**Supplemental Table 1.** Comparison of Neurotypical controls to SCA subtype. P-values are listed; * marks significant differences between the neurotypical control group and the SCA subtype compared.

|  | **Handedness** | **Gender** | **Age** | **IMCβγ** | **IMC_30-40_ – IMC_15-25_** |
| --- | --- | --- | --- | --- | --- |
| **SCA3** | 0.913 | 0.366 | 0.116 | 0.024* (0.024*) | 0.0009* (0.0018*) |
| **SCA6** | 0.206 | 0.425 | 0.002* | 0.355 (0.355) | 0.015* (0.03*) |

**Supplemental Table 2.** Comparison of SCA3 to SCA6 groups. P-values are listed; there were no significant differences between the SCA subtype for the metrics compared.

| **Handed-ness** | **Gender** | **Age** | **Disease duration** | **SARA score** | **IMCβγ** | **IMC_30-40_ – IMC_15-25_** |
| --- | --- | --- | --- | --- | --- | --- |
| 0.192 | 0.879 | 0.105 | 0.426 | 0.065 | 0.171 (0.342) | 0.464 (0.464) |

**Supplemental Table 3.** ROC area under the curve for differentiating the different groups based on IMC_βγ_.

| ***IMC_βγ_ AUC*** | **SCA-3** | **SCA-6** |
| --- | --- | --- |
| **Normal** | 0.715 | 0.586 |
| **SCA-3** | - | 0.634 |

**Supplemental Table 4.** ROC area under the curve for differentiating the different groups based on IMC_30-40_-IMC_15-25_.

| ***IMC_30-40_-IMC_15-25_ AUC*** | **SCA-3** | **SCA-6** |
| --- | --- | --- |
| **Normal** | **0.815** | 0.717 |
| **SCA-3** | - | 0.572 |

**
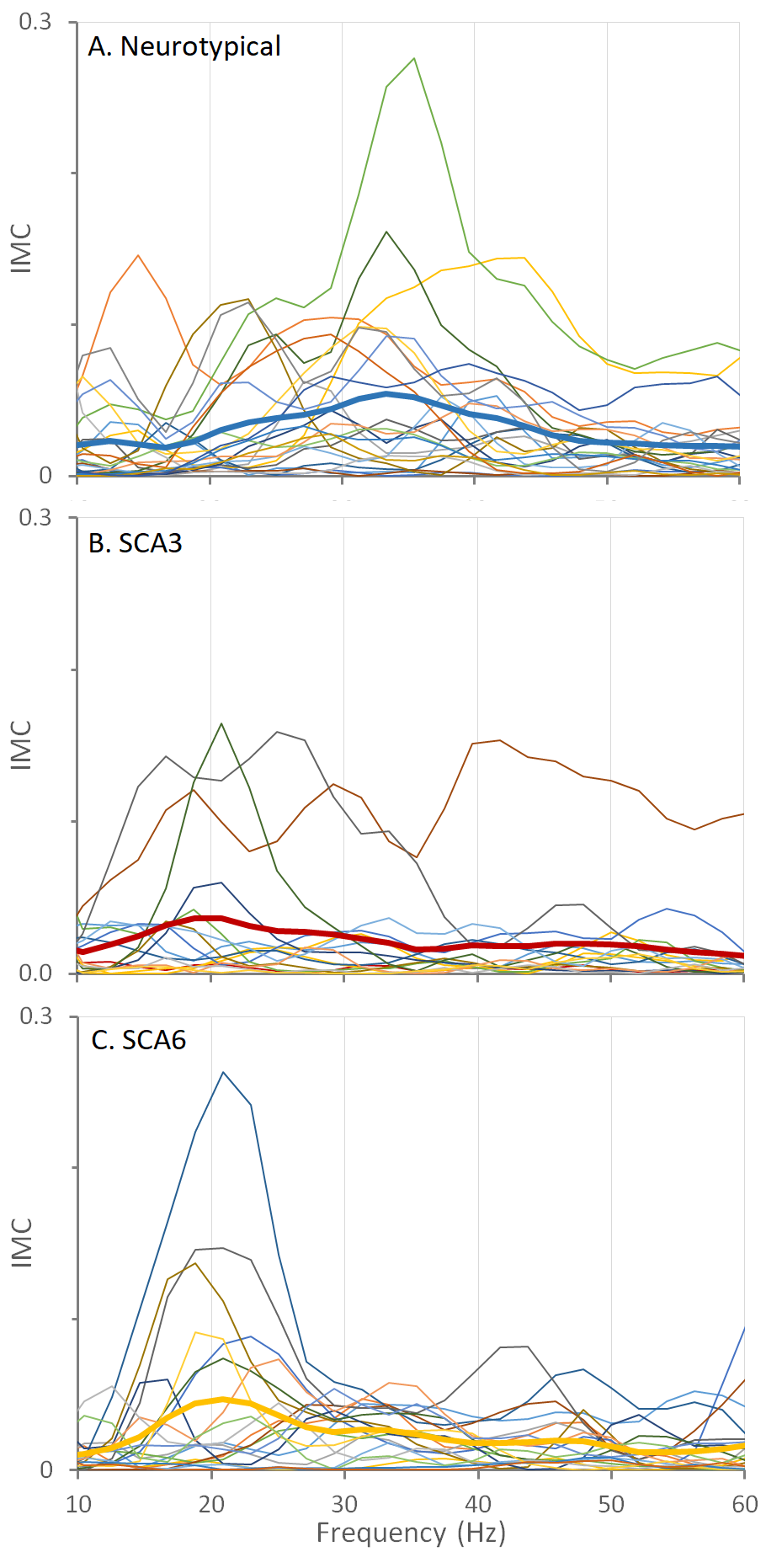
**

**Supplemental Figure.** IMC profiles from individual subjects and the average profile (thick lines) for each group. A. Neurotypical subjects. B. SCA3 subjects. C. SCA6 subjects.
